# Special Issue “Anticancer Drugs”

**DOI:** 10.3390/ph12030134

**Published:** 2019-09-16

**Authors:** Mary J. Meegan, Niamh M. O’Boyle

**Affiliations:** School of Pharmacy and Pharmaceutical Sciences, Trinity College Dublin, Trinity Biomedical Sciences Institute, 152-160 Pearse Street, 2 DO2R590 Dublin, Ireland

**Keywords:** snticancer drugs, cancer drug design, cancer immunotherapy, conjugate and hybrid drugs, cisplatin resistance, topoisomerase inhibitors, microtubule targeted drugs

## Abstract

The focus of this Special Issue of *Pharmaceuticals* is on the design, synthesis, and molecular mechanism of action of novel antitumor, drugs with a special emphasis on the relationship between the chemical structure and the biological activity of the molecules. This Special Issue also provides an understanding of the biologic and genotypic context in which targets are selected for oncology drug discovery, thus providing a rationalization for the biological activity of these drugs and guiding the design of more effective agents. In this Special Issue of *Pharmaceuticals* dedicated to anticancer drugs, we present a selection of preclinical research papers including both traditional chemotherapeutic agents and newer more targeted therapies and biological agents. We have included articles that report the design of small molecules with promising anticancer activity as tubulin inhibitors, vascular targeting agents, and topoisomerase targeting agents, alongside a comprehensive review of clinically successful antibody-drug conjugates used in cancer treatment.

We have great pleasure in accepting the invitation to be guest editors for this Special Issue of “Pharmaceuticals”. This volume presents reviews and original research papers by experts on a wide range of topics relevant to the topic of “Anticancer Drugs” and includes contributions relevant to both traditional chemotherapeutic agents and newer targeted therapies and biological therapeutics.

The global cancer burden is estimated by the World Health Organization at 18.1 million new cases and 9.6 million deaths in 2018 [1]. One in five men and one in six women worldwide are predicted to develop cancer during their lifetime, while one in 8 men and one in 11 women will die from the disease. The leading types of cancer worldwide in terms of the number of new cases are cancers of the lung and female breast; the largest number of deaths annually is from lung cancer (1.8 million deaths, 18.4% of the total), attributed to the poor prognosis for this cancer, followed by colorectal cancer (881,000 deaths, 9.2%), stomach cancer (783,000 deaths, 8.2%), and liver cancer (782,000 deaths, 8.2%).

This is an exciting era for cancer drug discovery and development and presents enormous opportunities for medicinal chemists, chemical biologists, and molecular biologists. In this Special Issue, we highlight both the opportunities and challenges available in the discovery and design of innovative cancer therapies, novel small-molecule cancer drugs, and antibody–drug conjugates. We hope to demonstrate the potential for future research in these areas. The transition from traditional cytotoxic chemotherapy to more targeted cancer drug discovery has resulted in an increasing selection of tools available to oncologists for cancer treatment. Continued research on the design of effective oncology drugs for application in chemotherapy has improved our understanding of the mechanism of the action of these drugs, expanded their activity/function spectrum, and unlocked new applications for improved patient outcomes. Chemotherapy is one of the most powerful tools available for treating cancer, and research continues to find new chemotherapy drugs, as well as new uses for existing ones. Newer types of drugs are being developed that attack cancer cells in different ways. These drugs include targeted therapies that are designed to attack cancer cells while demonstrating less damage to normal cells. Immunotherapies use the body’s own immune system to find and destroy cancer cells.

The development and availability of new effective oncology drugs is encouraging. The FDA (U.S. Food and Drug Administration) approved 8 drugs for orphan cancer indications in 2017 and 12 cancer drugs (26% of the total approvals), including 2 landmark approvals, to the first Chimeric Antigen Receptor (CAR)-T cell therapies. The approval of the IDH2 inhibitor enasidenib is also noteworthy, demonstrating that drugs can target cancer cells by blocking cancer-specific metabolic pathways. In 2018, the FDA approved 19 applications for new cancer drug and biologics, as well as 38 supplemental indications and 4 biosimilars [2]. In 2018, the FDA granted its second-ever approval for a ‘tissue-agnostic’ drug to treat tumors with a specific genetic change regardless of cancer type, and a second-ever biosimilar drug to treat cancer was approved. In 2018, 8 of 10 of the top selling pharmaceuticals were large molecules, and 6 out of 10 have cancer-related indications. Among the cancer drugs approved to date by the FDA in 2019 are selinexor, a first-in-class selective inhibitor of nuclear export for treating adult patients with relapsed or refractory multiple myeloma (RRMM). However, despite intense efforts and the discovery of many effective targeted therapies, oncology drug development remains challenging; combination therapy may be the future for oncology patients. 

It is recognized that cancer is a multifactorial disease and the genesis and progression of the disease are extremely complex. One of the major problems in the development of anticancer drugs is the emergence of multidrug resistance and relapse. Classical chemotherapeutic drugs directly target the DNA of the cell, but mutations enable the cell to develop resistance. More recent developments in the availability of anticancer drugs include molecular-targeted therapy such as targeting the proteins with abnormal expression inside the cancer cells, and the design and subsequent development of new anticancer small molecule agents. In recent years, many promising drug targets have been identified for effective exploitation in the treatment of cancer. Targeted chemotherapies are successful in certain malignancies; however the effectiveness has often been limited by drug resistance and side effects on normal tissues and cells. Their often high cost also precludes access to these agents by many patients who could potentially benefit. 

Many types of cancers are responsive to the “traditional” chemotherapy drug treatments, for example, alkylating agents, intercalating dugs, topoisomerase inhibitors, antimetabolites, and antimitotic drugs, as well as the more recently identified targeted therapies such as various kinase inhibitors. The targeted monoclonal antibodies have been proven to be spectacularly successful in specific cancers. A limited number of cancers can be completely cured using these treatment approaches. However, the success of cancer treatments varies enormously depending on the specific type of cancer diagnosed and stage of diagnosis.

Resistance exists against every effective anticancer drug and can develop by numerous mechanisms. Many patients exhibit intrinsic and acquired resistance to treatment with chemotherapeutic anticancer drugs and become refractory to treatment. Drug resistance can be caused by different mechanisms depending on the structure and action of the drug, including multi-drug resistance (MDR), cell death inhibition (apoptosis suppression), alterations in drug metabolism, epigenetic and drug targets, enhancement of DNA repair, and gene amplification. The development of MDR to chemotherapy remains a major challenge in treating cancer. 

With the development of genomic profiling technologies and selective molecular targeted therapies, the use of biomarkers plays an increasingly important role in the clinical management of cancer patients. To achieve a more comprehensive understanding of current research activities in the area of anticancer drugs, contributions of reviews and original research articles covering the different facets of anticancer drug research are now collected in this *Pharmaceuticals* Special Issue on “Anticancer Drugs”. The focus of this Special Issue is on the design, synthesis, and molecular mechanism of action of novel antitumor drugs and on the relationship between the chemical structure and biochemical reactivity of the molecules. This Special Issue provides an understanding of the biologic and genotypic context in which targets are selected for oncology drug discovery, thus allowing rationalization of the activity of these drugs and guiding the design of more effective agents. This Special Issue of *Pharmaceuticals* on “Anticancer Drugs” addresses a varied selection of preclinical research areas, including both traditional chemotherapeutic agents and newer more targeted therapies and biological agents. We have included articles describing the design of small molecules with promising anticancer activity as tubulin inhibitors, vascular targeting agents, and topoisomerase targeting agents, alongside a comprehensive review of antibody–drug conjugates. In addition, promising drug candidates under various phases of preclinical clinical trials are also described. Multi-acting drugs that simultaneously target different cancer cell signaling pathways may facilitate the design of effective anti-cancer drug therapies. The specific topics include synthesis and evaluation of novel small molecules targeting biomolecules such as tubulin and topoisomerase; development of novel nanocarrier drug delivery systems for cytotoxic cisplatin, cisplatin resistance in oesophageal cancer, approaches to treatment of 5-fluorouracil-induced intestinal mucositis; mechanism of action of the anti-prostate cancer drug abiraterone; a study of [^18^F]FDG-PET/CT in clinical oncology; cyclooxygenase-1 (COX-1) and COX-1 inhibitors in cancer; and chemistry and clinical implications of antibody–drug conjugates for cancer therapy. 

Chemotherapy is widely used to treat cancer, which is the second leading cause of death worldwide. Nonspecific distribution and uncontrollable release of drugs in conventional drug delivery systems have led to the development of smart nanocarrier-based drug delivery systems, which are also known as smart drug delivery systems (SSDS) as an alternative to chemotherapy. SDDSs can deliver drugs to the target sites with reduced dosage frequency and in a controlled manner to reduce the side effects experienced in conventional drug delivery systems. Makharza et al. describe selective delivery of the widely used chemotherapeutic drug cisplatin to glioblastoma U87 cells by the design of a hybrid nanocarrier composed of magnetic γ-Fe_2_O_3_ nanoparticles and nanographene oxide [3]. They demonstrated negligible toxicity for the nanoparticle system; the anticancer activity of cisplatin was retained with loading onto the carrier, together with control of drug delivery at the target site. Although cisplatin is one of the most widely used chemotherapeutic drugs for the treatment of solid tumors, the development of resistance hinders the success of this drug in the clinic.

The study by Buckley et al. provides novel insights into the molecular and phenotypic changes in an isogenic oesophageal adenocarcinoma model of acquired cisplatin resistance in oesophageal adenocarcinoma [4]. Key differences that could be targeted to overcome cisplatin resistance are identified in this study, including differences in treatment sensitivity, gene expression, inflammatory protein secretions, and metabolic rate in their model. It is of interest that cisplatin resistant cells have an altered metabolic profile under normal and low oxygen conditions. The molecular differences identified in this study, for example, increased sensitivity to radiation and 5-fluorouracil of cisplatin resistant cells, provide novel insight into cisplatin resistance in oesophageal adenocarcinoma. The authors have identified potential molecular processes that could be targeted to overcome cisplatin resistance and improve therapeutic outcomes for oesophageal adenocarcinoma patients. 

Even with the emergence of targeted therapies for cancer treatment, natural products and their derivatives that target microtubules are some of the most effective drugs used in the clinical treatment of solid tumors and hematological malignancies. Many natural products have been discovered that bind to tubulin/microtubules and disrupt microtubule function. Although these drugs inhibit mitosis, emerging evidence indicates that their actions are complex and inhibit signaling events important for carcinogenesis. Microtubule-targeted drugs are essential chemotherapeutic agents for various types of cancer, for example, taxol and the vinca alkaloids such as vincristine and vinblastine. The design and evaluation of novel small molecules that target mitosis continues to attract the interest of medicinal chemists. Microtubules are an important target for structurally diverse natural products, and a fuller understanding of the mechanisms of action of these drugs will promote their optimal use. A series of 3-vinyl-β-lactams (2-azetidinones) was designed, synthesized, and evaluated as potential tubulin polymerization inhibitors in breast cancer cells by Wang et al [5]. The compounds inhibited the polymerization of tubulin, and were shown to interact at the colchicine-binding site on tubulin, resulting in significant G_2_/M phase cell cycle arrest and mitotic catastrophe. These compounds are promising candidates for development as antiproliferative microtubule-disrupting agents. Continued efforts to identify the effects of microtubule targeting agents on oncogenic signaling pathways will provide opportunities to discover therapeutic uses for these drugs.

Ellipticines have well documented anticancer activity, based on the structure of the alkaloid ellipticine, which inhibits the enzyme topoisomerase II via intercalative binding to DNA in particular. The anti-tumor alkaloid ellipticine and its derivatives act as potent anticancer agents via a combined mechanism involving cell cycle arrest and induction of apoptosis. The prevalent DNA-mediated mechanisms of anti-tumor, mutagenic, and cytotoxic activities of ellipticine are DNA intercalation, inhibition of DNA topoisomerase II activity, and covalent binding to DNA. However, owing to limitations in synthesis and coherent screening methodology, it has not been possible to achieve the full structure-activity relationship (SAR) profile of this important anticancer class to date. Miller et al. have addressed this issue, and have explored the anticancer activity of this potent natural product by a series of substitutions on the heterocyclic structure [6]. The synthesis of a panel of novel 11-substituted ellipticines is described, with two specific derivatives showing potency and diverging cellular growth effects on cancer cell lines on a panel of 60 National Cancer Institute (NCI) cell lines.

Side effects of chemotherapy can limit its usefulness. Intestinal mucositis is a common complication associated with 5-fluorouracil (5-FU) treatment, a chemotherapeutic agent used for colon, oesophageal, stomach, breast, pancreatic, and cervical cancers. Miranda et al. have evaluated the effects of Cashew gum (bark exudate from *Anacardium occidentale* L.) as a potent anti-inflammatory agent on experimental intestinal mucositis induced by 5-FU [7]. Use of Cashew gum, as a versatile polymer scaffold material in formulating pharmaceuticals, is of considerable interest owing to the polymer’s biocompatibility, low toxicity, and biodegradability. The authors report that Cashew gum prevented 5-FU-induced histopathological changes and decreased oxidative stress through decrease of malondialdehyde levels and increase of glutathione concentration. The authors suggest that Cashew gum reverses the effects of 5-FU-induced intestinal mucositis. Cashew gum decreases inflammation, oxidative stress, and intestinal injury induced by 5-fluorouracil in the duodenum. The effects of Cashew gum were found to be related to the cyclooxygenase-2 (COX-2) pathway. Cashew gum attenuated an inflammatory process by decreasing myeloperoxidase activity, intestinal mastocytosis, and interleukin (IL)-1β and cyclooxygenase-2 (COX-2) expression. The co-administration of Cashew gum and celecoxib completely reversed COX-2 and IL-1β expression and the intestinal injury induced by 5-FU. It is suggested that Cashew gum has potential application in the development of novel treatments for intestinal mucositis owing to 5-FU and other antineoplastic agents. 

Knowledge about the specificity of the cytochrome P450 CYP17A1 enzyme activities is of importance for the development of treatments for the polycystic ovary syndrome and inhibitors for prostate cancer therapy. Androgens have an important role in the development of both normal prostate epithelium and prostate cancer and variants of genes involved in androgen metabolism may be linked to an increased risk of prostate cancer. Cytochrome P450 17α-hydroxylase/17,20-lyase (CYP17A1) is a key regulatory enzyme in the steroid metabolism; it catalyses both 17,20-lyase and 17α-hydroxylase transformations and is essential for the biosynthesis of androgens and glucocorticoids. Fernández-Cancio et al. discuss the mechanism of the dual activities of human CYP17A1 and the interaction of this enzyme with the anti-prostate cancer drug abiraterone [8]. These results are presented in their studies of a novel V366M mutation causing 17,20 lyase deficiency. Molecular dynamics simulations are effectively used to demonstrate how the V366M mutation facilitates a mechanism for dual activities of human CYP17A1 requiring the conversion of pregnenolone to 17OH-pregnenolone, which re-enters the active site for conversion to dehydroepiandrosterone. The effectiveness of the anti-prostate cancer drug abiraterone as a potent inhibitor of CYP17A1 is rationalized. 

Positron emission tomography (PET) is a functional imaging modality widely used in clinical oncology. Over the years, the sensitivity and specificity of PET has improved with the advent of specific radiotracers, increased technical accuracy of PET scanners, and incremental experience of radiologists. The potential influence of individual molecular markers of glucose transport, glycolysis, hypoxia, and angiogenesis, in addition to the relationships between these key cellular processes and their influence on fluorodeoxyglucose (FDG) uptake, is reviewed by O’Neill et al [9]. The potential role for biomolecular profiling of individual tumors to predict positivity on ^18^F-fluorodeoxyglucose (^18^F-FDG) positron emission tomography/computed tomography (PET/CT) imaging is discussed with a view of enhancing accuracy and clinical utility. 

Cancer may originate in the chronic inflammation setting associated with persistent infections, immune-mediated damage, or prolonged exposure to irritants. Prostaglandins and thromboxane are lipid signalling molecules produced from arachidonic acid by the action of the cyclooxygenase isoenzymes COX-1 and COX-2. Pannunzio and Coluccia review the role of cyclooxygenase-1 (COX-1) and cyclooxygenase-2 (COX-2) inhibitors in cancer [10]. The role of cyclooxygenases (particularly COX-2) and prostaglandins (particularly PGE₂) in cancer-related inflammation has been extensively investigated. Although COX-1 expression increases in several human cancers, the contribution of COX-1 remains much less explored. COX-1 and COX-2 isoforms seem to operate in a coordinated manner in cancer pathophysiology. In some cases, such as serous ovarian carcinoma, COX-1 plays a significant role. The precise genetic and molecular defects underlying epithelial ovarian cancer remain largely unknown, and treatment options for patients with advanced disease are limited. Human epithelial ovarian tumors have increased levels of COX-1, but not COX-2. The authors discuss the choice of the most appropriate tumor cell models for investigation of the role of COX-1 in the context of arachidonic acid metabolic network and review the in vitro and in vivo antitumor properties of COX-1-selective inhibitors. 

Antibody–drug conjugates (ADCs) are highly targeted biopharmaceutical drugs that combine monoclonal antibodies specific to surface antigens present on particular tumour cells with highly potent anti-cancer agents linked *via* a chemical linker. ADCs have become a powerful class of therapeutic agents in oncology and hematology, with five approved drugs on the market, namely, Ado-trastuzumab emtansine, Brentuximab Vedotin, Gemtuzumab Ozogamicin, Inotuzumab Ozogamicin, and Polatuzumab Vedotin-piiq. This targeted approach can improve the tumour-to-normal tissue selectivity and specificity in chemotherapy. The continuing developments in the therapeutic use of antibody-drug conjugates for cancer therapy is discussed by Dan et al., where they consider the chemistry aspects of the conjugate design and stability together with the drug-linker targeting [11]. This review focuses on site-specific conjugation methods for producing homogenous ADCs with a constant drug–antibody ratio (DAR) and discusses the major challenges in conventional conjugation methods. 

The past forty years have seen major developments in the understanding of the cellular and molecular biology of cancer. Significant increases have been achieved in long-term survival for many cancers, such as the use of tamoxifen touted as one of the game-changers for breast cancer, treatment of chronic myeloid leukemia with imatinib, and the success of biological drugs. The overall success rate for oncology drugs in clinical development estimated at ~10%, while the cost in introducing a new drug to market is estimated at greater than 1 billion US$. A number of factors are to be considered in the development of effective anticancer drugs. These include the role of the target identified in the pathogenesis of specific human cancers, target overexpression in a specific malignancy, interactions among the cellular components of malignant tissues, choice of preclinical cancer models of drug effects, balance between drug safety and efficacy in cancer patients, and the benefits of biomarkers in achieving a personalized approach to cancer drug development [12]. Unfortunately, resistance to treatment continues to be challenging, and contributes to mortality and morbidity. However, as is evident from the research and review papers presented in this *Pharmaceuticals* Special Issue on “Anticancer Drugs”, significant efforts are being made to develop and improve cancer treatments and to translate basic research findings into clinical use, resulting in improvements in survival rates and quality of life for cancer patients. 
